# Cold Water Immersion Improves the Recovery of Both Central and Peripheral Fatigue Following Simulated Soccer Match-Play

**DOI:** 10.3389/fphys.2022.860709

**Published:** 2022-08-15

**Authors:** Mustapha Bouchiba, Nicola Luigi Bragazzi, Slim Zarzissi, Mouna Turki, Firas Zghal, Mohamed Amine Grati, Wael Daab, Fatma Ayadi, Haithem Rebai, Hassen Ibn Hadj Amor, Thomas J. Hureau, Mohamed Amine Bouzid

**Affiliations:** ^1^ Research Laboratory, Education, Motricité, Sport et Santé, EM2S, LR19JS01, High Institute of Sport and Physical Education, University of Sfax, Sfax, Tunisia; ^2^ Laboratory for Industrial and Applied Mathematics (LIAM), Department of Mathematics and Statistics, York University, Toronto, ON, Canada; ^3^ Laboratory of Biochemistry, CHU Habib Bourguiba, Sfax University, Sfax, Tunisia; ^4^ Faculté des Sciences du Sport, Université Côte d'Azur, Nice, France; ^5^ Université de Monastir Faculté de Médecine de Monastir, Monastir, Tunisia; ^6^ Oxidative Stress and Muscular Protection Laboratory (UR3072), Faculty of Medicine, Mitochondria, University of Strasbourg, Strasbourg, France; ^7^ European Centre for Education, Research and Innovation in Exercise Physiology (CEERIPE), Faculty of Sport Sciences, University of Strasbourg, Strasbourg, France

**Keywords:** neuromuscular fatigue, recovery strategy, football, cold water immersion, exercise performance

## Abstract

The present study aimed to investigate the effect of cold water immersion (CWI) on the recovery of neuromuscular fatigue following simulated soccer match-play. In a randomized design, twelve soccer players completed a 90-min simulated soccer match followed by either CWI or thermoneutral water immersion (TWI, sham condition). Before and after match (immediately after CWI/TWI through 72 h recovery), neuromuscular and performance assessments were performed. Maximal voluntary contraction (MVC) and twitch responses, delivered through electrical femoral nerve stimulation, were used to assess peripheral fatigue (quadriceps resting twitch force, Q_tw,pot_) and central fatigue (voluntary activation, VA). Performance was assessed via squat jump (SJ), countermovement jump (CMJ), and 20 m sprint tests. Biomarkers of muscle damages (creatine kinase, CK; Lactate dehydrogenase, LDH) were also collected. Smaller reductions in CWI than TWI were found in MVC (-9.9 ± 3%*vs*-23.7 ± 14.7%), VA (-3.7 ± 4.9%*vs*-15.4 ± 5.6%) and Q_tw,pot_ (-15.7 ± 5.9% vs*.* -24.8 ± 9.5%) following post-match intervention (*p* < 0.05). On the other hand, smaller reductions in CWI than TWI were found only in Q_tw,pot_ (-0.2 ± 7.7% vs*.* -8.8 ± 9.6%) at 72 h post-match. Afterwards, these parameters remained lower compared to baseline up to 48–72 h in TWI while they all recovered within 24 h in CWI. The 20 m sprint performance was less impaired in CWI than TWI (+11.1 ± 3.2% vs*.* +18 ± 3.6%, *p* < 0.05) while SJ and CMJ were not affected by the recovery strategy. Plasma LDH, yet no CK, were less increased during recovery in CWI compared to TWI. This study showed that CWI reduced both central and peripheral components of fatigue, which in turn led to earlier full recovery of the neuromuscular function and performance indices. Therefore, CWI might be an interesting recovery strategy for soccer players.

## 1 Introduction

During a single match-play, soccer players cover 10–13 km, of which 2–3 km at high-intensity running involving accelerations, decelerations, direction changing and tackles (Mohr et al., 2003). As a consequence of this physical demand, a soccer match-play generates substantial neuromuscular fatigue (Brownstein et al., 2017; Thomas et al., 2017) with implications for subsequent performance (Andersson et al., 2008; Ispirlidis et al., 2008).

Neuromuscular fatigue can be defined as a transient reduction of the muscle’s ability to generate force and is related to mechanisms of central and/or peripheral origins (Gandevia, 2001). Central fatigue is defined as a failure of the central nervous system to voluntarily activate the muscle (Gandevia, 2001), while peripheral fatigue is associated to the biochemical changes within the active muscle (Allen et al., 2008). Interestingly, studies performed during simulated (Thomas et al., 2017) or competitive (Brownstein et al., 2017) soccer match-play showed that players experienced both central and peripheral fatigue following a match, which persisted for up to 72 h after match completion. Similarly, markers of muscle damage and inflammation increased after a soccer match-play in elite players, with a return to baseline values after 72 h (Ascensão et al., 2008; Ispirlidis et al., 2008). These physiological impairments observed following a soccer match-play led to a transient decrease in physical performance as evidenced by several indices such as impairments in measures of jump and sprint ability and force production (Andersson et al., 2008; Ispirlidis et al., 2008). Here again, physical performance was not restored quickly and remained lower than baseline for up to 72 h (Ispirlidis et al., 2008; Brownstein et al., 2017).

Nowadays, the competitive schedule is heavy for elite soccer players, with up to two matches per week over the course of a 9-months season, inevitably leading to short recovery periods (∼3–4 days) between matches (Nédélec et al., 2012). Consequently, players are likely to demonstrate residual fatigue at the start of congested matches, with a negative effect on physical performance and a substantial increase in injury risk (Dupont et al., 2010). Therefore, the need to accelerate the recovery process after soccer match-play seems vital for performance. With this in mind, the use of cold water immersion (CWI) after exercise might be an interesting strategy, as it has been proven to reduce muscle damage and inflammation immediately following a soccer match (Bailey et al., 2007; Bouzid et al., 2018) and to result in faster recovery of physical performance in professional soccer players (Abaïdia et al., 2017; Bouzid et al., 2018).

Although there is compelling evidence that CWI enhances post-exercise recovery, its mechanistic consequences on neuromuscular fatigue and recovery are still unknown (Ihsan et al., 2016). More specifically, since soccer match-play induces, as described above, neuromuscular fatigue of both central and peripheral origins (Brownstein et al., 2017; Thomas et al., 2017), it is important to characterize the acute and delayed effects of CWI on these two fatigue components.

Thus, the aim of the present study was to examine the impact of immediate post-exercise CWI on the recovery time-course of neuromuscular fatigue following a simulated soccer match-play. We hypothesized that CWI would lead to lower peripheral and central fatigue development, and therefore faster recovery kinetics, compared to a sham, thermoneutral water immersion (TWI).

## 2. Methods

### 2.1. Participants

Upon ethical approval from the local clinical Research Ethics Committee (CPP N° 0104), 12 male soccer players volunteered to participate in this study (mean ± SD; age: 22.9 ± 0.9 years; body mass: 71.3 ± 6.4 kg; height: 1.84 ± 0.06 cm, Maximal aerobic speed: 16.4 ± 2.3 km/h, MVC: 829.3 ± 165.5 N, SJ: 36.6 ± 1.94 cm, CMJ: 37.5 ± 3.7 cm, 20 m sprint: 2.8 ± 0.37s). All participants were playing in the third Tunisian National League and trained at least 5 days per week for∼1.5 h per day during the competitive period. Experimental testing sessions took place in the late off-season to early pre-season phase of the players’ training year, while players were still resting.

### 2.2. Experimental Design

#### 2.2.1. Overview

**FIGURE 1:**
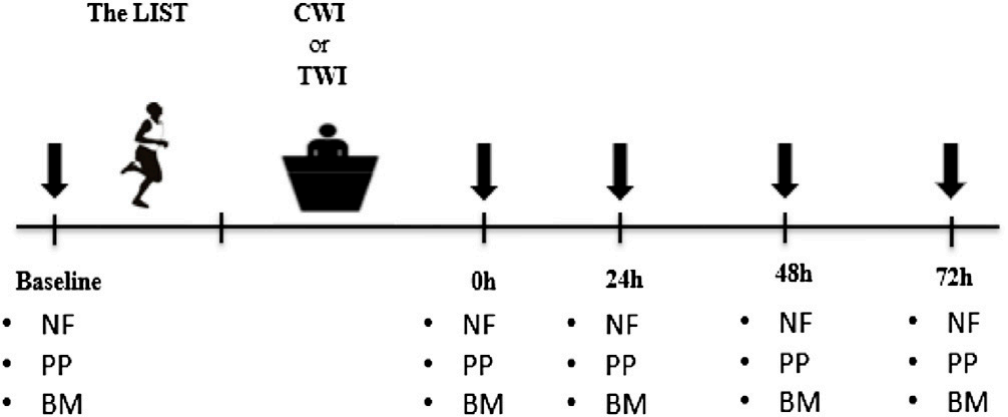
Overview of the entire experimental period. Downward arrows denote the time points when neuromuscular, physical and biochemical parameters were recorded. The LIST: Loughborough Intermittent Shuttle Test. CWI: Cold water immersion; TWI: Thermoneutral water immersion. NF: Neuromuscular function; PP: Physical performance; BM: Blood markers.

All participants completed two preliminary visits to ensure familiarization with the measurements implemented in this study, and another visit to determine the maximal aerobic speed via a VAMEVAL incremental running test (Uger and Boucher, 1980). Next, participants completed two experimental sessions, separated by at least seven days in randomized order, where they performed a simulated soccer match followed by 10 min of recovery using thermoneutral water immersion (TWI) or cold-water immersion (CWI) (Figure 1). For each condition, blood biomarkers, neuromuscular function and physical performance assessments were performed at baseline, immediately after CWI/TWI, and 24, 48 and 72 h after the match to ascertain the time-course of recovery of all variables of interest. All measures of physical function and blood markers were performed following the neuromuscular assessment and the completion of a standardized warm-up. All assessments were performed on an indoor synthetic track, in a quiet laboratory under standardized conditions (temperature: 21–23°C; relative humidity: 40–60%).

#### 2.2.2. Simulated Soccer Match

To ensure an accurate and consistent simulation of the physical demands of a soccer match-play throughout the scheduled experimental visits, participants performed the Loughborough Intermittent Shuttle Test (LIST) (Nicholas et al., 2000). As described by Nicholas et al. (2000), the LIST comprises two parts. Part A is of fixed duration and consists of 5 × 15-min exercise periods interspersed by a 3-min recovery period. The exercise sequences were designed in the following order:

• 3 × 20 m walking
• 1 × 20 m at maximal running speed
• 4 s recovery
• 3 × 20 m running at 55% of VO2max
• 3 × 20 m at 95% of VO2max

Part B of the test is an open-ended period of intermittent shuttle running. The participants run continuously between the 20 m markers, alternating between speeds of 55% and 95% of VO_2max_ every 20 m. This part is designed to last approximately 10 min, and continues until they are unable to maintain the required speed for two consecutive shuttles. During the test, players were informed about their running and walking speed every 20 m using audible signals issued by a software developed for this purpose. Heart rate was continuously recorded throughout the simulated match using heart rate monitors (Polar Team System, Polar electro, Kempele, Finland). The LIST was performed in artificial grass.

#### 2.2.3 Cold and Thermoneutral Water Immersion

Immediately after completion of the simulated match-play, participants underwent a single 10-min bout of lower limb water immersion at cold (10 ± 2°C) or thermoneutral (28 ± 2°C) exposure. Participants were seated, with a 90° angle between torso and legs, in a bath filled to the level of the superior iliac crest to assure immersion of the entire lower limb. A thermometer (212–130, RS Products, Texas, USA) was used to keep track of water temperature. When necessary, experimenters added cubed ice into water to maintain the required temperature.

### 2.3 Data Collection and Analysis

#### 2.3.1 Assessment of Neuromuscular Function of the Quadriceps

Participants were seated on an isometric dynamometer (Good Strength, Metitur, Finland) equipped with a cuff attached to a strain gauge. This cuff was adjusted 2 cm above the lateral malleolus using a non-compliant Velcro strap for continuous recording of quadriceps force. Participants were stabilized using safety belts strapped across the chest, thighs and hips, in order to avoid lateral, vertical or frontal displacements. Subjects were seated with a 90° knee flexion angle from full extension. A constant current stimulator (Digitimer Limited, Hertfordshire, United Kingdom) delivered single square-wave stimuli of 1 ms duration with maximal voltage of 400 V percutaneously over the femoral nerve. The cathode (self-adhesive electrode: Ag-AgCl, 10-mm diameter) was positioned firmly on the femoral triangle. The anode, a 10 × 5 cm self-adhesive stimulation electrode (Compex Medical SA, Ecublens, Switzerland) was placed midway between the greater trochanter and the iliac crest. Optimal stimulation intensity was determined from M-wave and force measurements before each testing session. The stimulation intensity was increased by 5 mA until there was no further increase in peak twitch force (i.e., plateau in knee extensor twitch force) and concomitant VL, VM, and RF peak-to-peak maximal M-wave amplitude (M_max_). During the subsequent testing procedures, the intensity was set to 150% of this intensity (supramaximal intensity) to avoid the potential confounding effect of axonal hyperpolarization (Burke, 2002). The neuromuscular assessment began with two practices of MVCs to ensure potentiation of subsequent evoked measures, followed by three ∼3 s MVCs, all separated by 30 s. For each MVC, two electrical nerve stimulations were delivered over the femoral nerve. The first stimulation, delivered during the MVC, was named the superimposed twitch, and the second stimulation, delivered 3 s after the MVC, was named the potentiated twitch (Q_tw,pot_). This 3-s delay allowed for the obtaining of a potentiated mechanical response which reduces the variability of the measurement compared to a non-potentiated twitch (Kufel et al., 2002). The average of the three MVCs was included in the analysis. Quadriceps (peripheral) fatigue was calculated as the difference in Q_tw,pot_ from baseline to post exercise (∆Q_tw,pot_). ‘Global’ (peripheral + central) fatigue was determined as the difference in MVC from baseline to post exercise (∆MVC, %). For central fatigue, exercise-induced changes in voluntary activation (VA) were calculated using both superimposed and potentiated twitches amplitude as follows (Merton, 1954):

$$\mathrm{VA}\left(\%\right)=\left[1-\left(\mathrm{Superimposed}\;\mathrm{twitch}/Q_{\mathrm{tw,pot}}\right)\right]\times 100$$

#### 2.3.2 Electromyography

The EMG signals were recorded using bipolar silver chloride surface electrodes (Dormo Electrodes, Ag-AgCl, SX-50 ECG, Spain). The recording electrodes were taped lengthwise to the skin over the muscle belly in accordance with SENIAM recommendations (Hermens et al., 1999), with an inter-electrode distance of 20 mm. The reference electrode was attached to the patella. Low impedance (Z < 5 kΩ) at the skin–electrode surface was obtained through shaving, abrading the skin with emery paper and cleaning it with alcohol. The position of the electrodes was marked with indelible ink to ensure identical placement at subsequent visits. Electromyographic signals were amplified (Octal Bio Amp ML 138, ADInstruments, Australia) with a band with frequency ranging from 10 Hz to 1 kHz (common mode rejection ratio >96 dB, gain = 1,000) and simultaneously digitized together with force signals using an acquisition card (Powerlab 16SP, ADInstruments, Australia) and the Labchart 7.0 software (ADInstruments, Australia). The sampling frequency was 2 kHz. For each Q_tw,pot_, peak-to-peak amplitude of the maximal muscle action potential (i.e. M-wave, M_max_) was measured. During MVC trials, muscle activation of VL, VM and RF were calculated with the root mean square (RMS) on EMG signals, and added together. Specifically, the RMS was determined over a fixed 1-s window starting 500 ms before peak force.

#### 2.3.3 Assessment of Physical Performance

Physical performance was assessed using the three following tests: squat jump (SJ), countermovement jump (CMJ) and 20 m sprint. Participants performed three trials for SJ and CMJ, interspersed by1-min passive recovery periods. The best of three trials in each test was recorded for a later analysis. Jump height was measured using an optical timing system (Optojump, Microgate, Milan, Italy). For the 20 m sprint, two trials, interspersed by 1-min passive recovery, were performed. The better of the two trials was recorded for later analysis. Performance time was measured using photoelectric cells placed at 0 and 20 m (Brower Timing System, IRD-T175, Draper, UT, USA).

#### 2.3.4 Blood Biomarkers

Venous blood samples (∼5 ml) were drawn from an antecubital vein into an EDTA tube. All extracted blood samples underwent a blood fractionation process. Whole blood was centrifuged at 1500 rpm for 15 min at 4°C, and then separated into its component parts in order to obtain plasmatic fractions. Subsequently, samples were stored at −70°C until analyzed. Plasma creatine kinase (CK) and lactate dehydrogenase (LDH) activity were measured using spectrophotometric test kits (Myoglobin bioMerieux A11A01632, Horiba-ABX, Montpellier, France). Intra-and inter-assay coefficients of variation were 0.3%and1.4% respectively.

### 2.4 Statistical Analyses

Normality of every dependent variable and homogeneity of distribution variances (equal variance) were confirmed using Shapiro-wilk test and the Levene test, respectively. Two-way ANOVA (Condition [CWI or TWI] × Time [Baseline,post-match, 24h, 48h and 72 h recovery]) with repeated measures was used to test differences in neuromuscular function, physical performance, and enzymes activity. When a significant difference was found, multiple comparison analysis was performed with Bonferroni post hoc test. Effect sizes were reported as Cohen’s *d.* Data presented in the results section are expressed as mean ± standard deviation and were analyzed using Statistica for Windows software (version 10, StatSoft, Inc, Tulsa, OK). Statistical significance was set at *p* < 0.05.

## 3 Results

### 3.1 Neuromuscular Fatigue and Recovery

**FIGURE 2:**
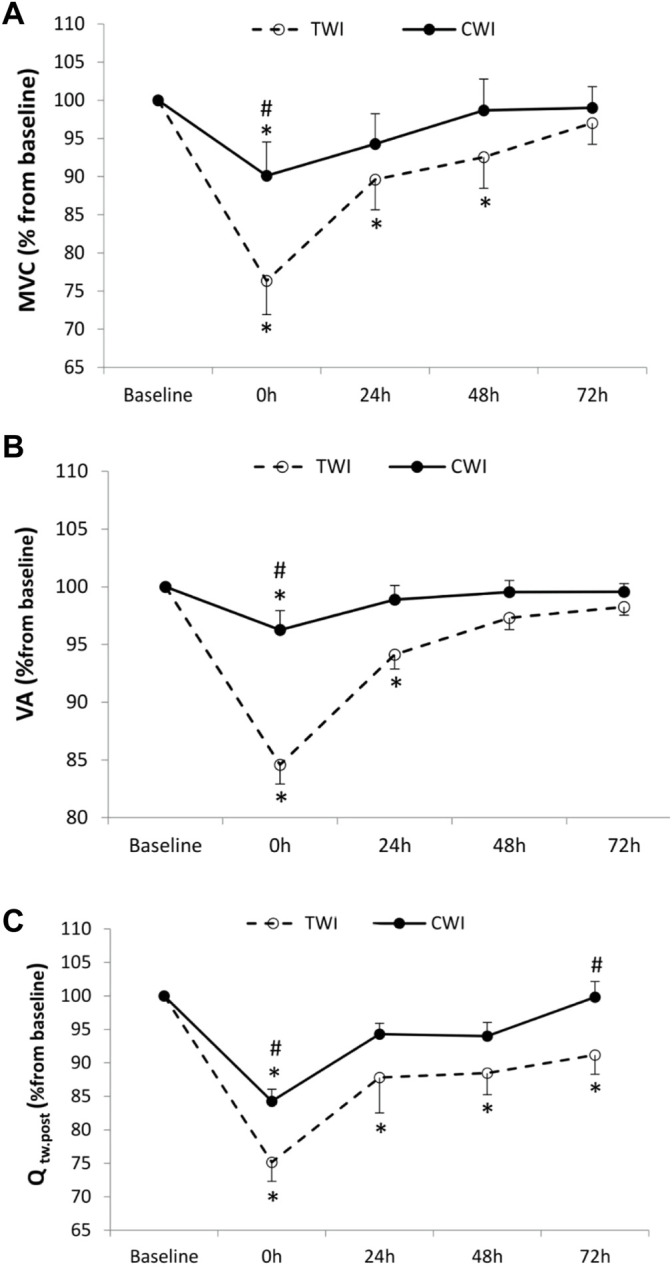
Maximal voluntary contraction (MVC) **(A)**, Voluntary activation (VA) **(B)**, and quadriceps potentiated twitch force (Q_tw,pot_) **(C)** values measured at baseline and following the match (post, 24h, 48h and 72 h) after cold water immersion (CWI) or thermoneutral water immersion (TWI). *Significant difference in comparison to baseline (*p* < 0.05). ^#^Significant difference in comparison to TWI (*p* < 0.05).

The decrease in MVC from baseline (100%) to immediately post-match intervention (i.e., fatigue) was significantly lower (*p* < 0.001, *d =* 1.3) in CWI (-9.9 ± 3%) compared to TWI (-23.7 ± 14.7%). Later, MVC recovered and returned to baseline values at 24 h in CWI and at 72 h in TWI (Figure 2A). The decrease in VA from baseline (100%) to post-match was significantly lower (*p* < 0.001, *d =* 1.3) in CWI (-3.7 ± 4.9%) compared to TWI (-15.4 ± 5.6%). After that, VA recovered and returned to baseline values at 24 h in CWI and at 48 h in TWI (Figure 2B). The decrease in Q_tw,pot_ from baseline (100%) to post-match was significantly lower (*p* < 0.05, *d =* 1.15)in CWI (-15.7 ± 5.9%) compared to TWI (-24.8 ± 9.5%). Also, the decrease in Q_tw,pot_ from baseline to 72 h was significantly lower (*p* < 0.05, *d =* 0.98) in CWI (-0.2 ± 7.7%) compared to TWI (-8.8 ± 9.6%). Then, Q_tw,pot_ recovered and returned to baseline values at 24 h in CWI, yet, it did not fully recover in 72 h in TWI (Figure 2C). Maximum M-wave (VL, ∼9.44 ± 0.6 mV, *p* > 0.05, VM, ∼ 6.22 ± 0.8mV, *p* > 0.05, RF, ∼ 5.3 ± 0.4 mV, *p* > 0.05) and RMS/M_max_ (∼0.05 ± 0.003 mV, *p* > 0.05) did not differ from baseline values throughout recovery period in both conditions.

### 3.2 Physical Performance

**FIGURE 3:**
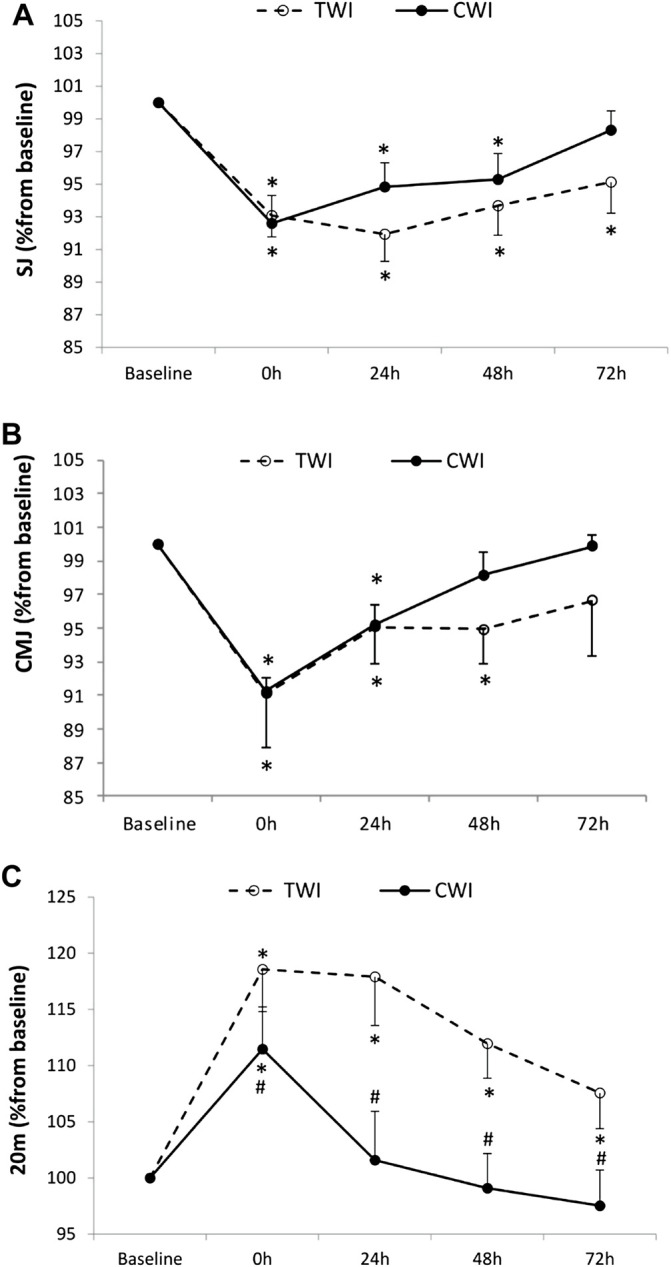
Squat jump (SJ) **(A)**, countermovement jump (CMJ) **(B)**, and 20 m sprint **(C)** values measured at baseline and following the match (post, 24h, 48h and 72 h) after cold water immersion (CWI) or thermoneutral water immersion (TWI). *Significant difference in comparison to baseline (*p* < 0.05). ^#^Significant difference in comparison to TWI (*p* < 0.05).

The decrease in SJ from baseline (100%) to post-match was significant, yet not different between conditions (CWI, -7.4 ± 5.7%; TWI,-6.9 ± 4.3%, *p* > 0.05). Afterwards, SJ recovered and returned to baseline values at 72 h in CWI but did not fully recover in 72 h in TWI (Figure 3A). The decrease in CMJ from baseline (100%) to post-match was significant, however, not different between conditions (CWI, -8.8 ± 2.8%; TWI, -8.9 ± 10.7%, *p* > 0.05). Posteriorly, CMJ recovered and returned to baseline values at 48 h in CWI and at 72 h in TWI (Figure 3B). The increase in 20 m time from baseline (100%) to post-match was significantly lower (*p* < 0.05, *d =* 2.17) in CWI (+11.1 ± 3.2%) compared to TWI (+18.5 ± 3.6%). Then, 20 m performance recovered and returned to baseline values at 24 h in CWI but did not fully recover in 72 h in TWI (Figure 3C).

### 3.3 Blood Biomarkers

**FIGURE 4:**
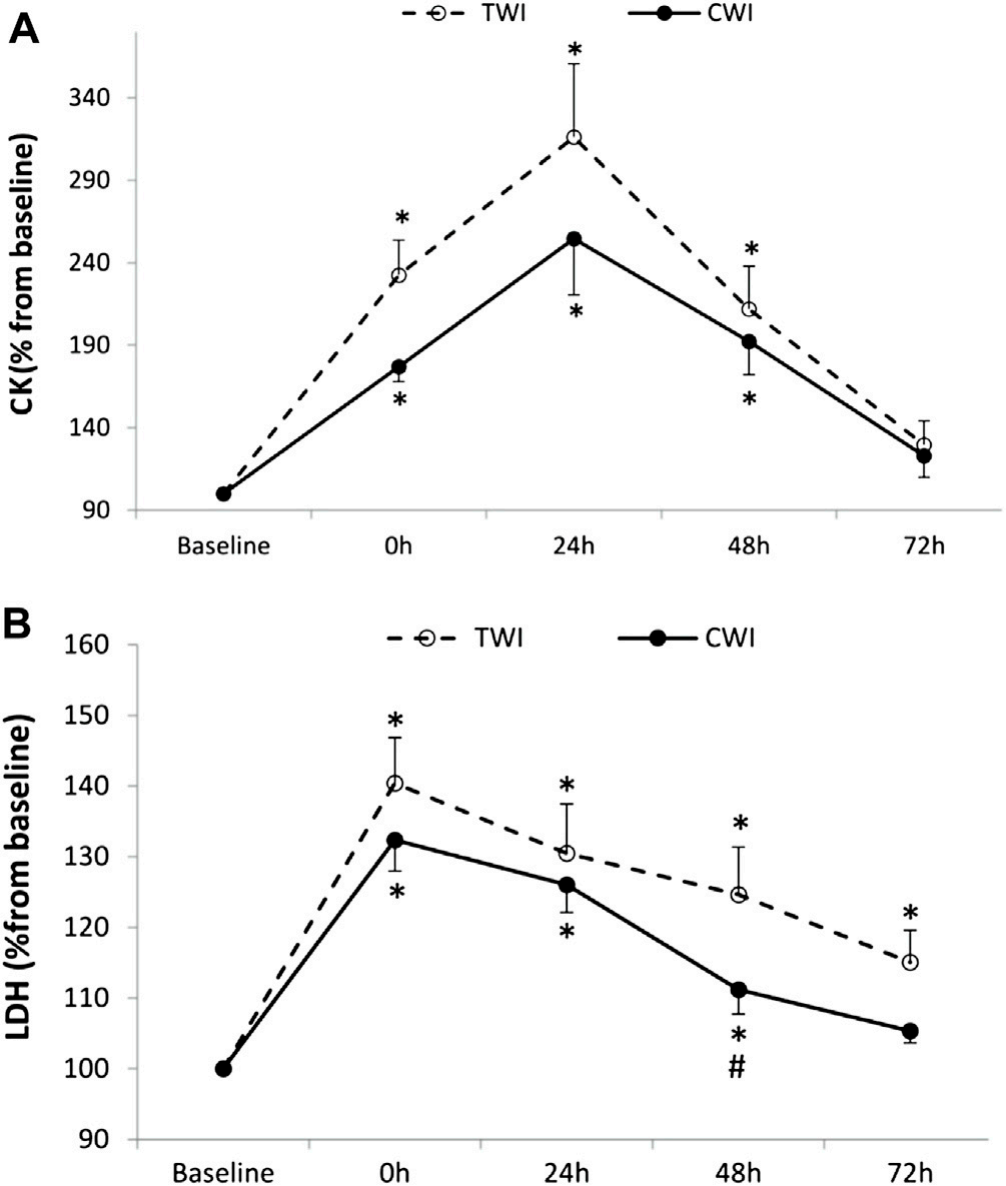
Plasma creatine kinase (CK) **(A)** and Lactate dehydrogenase (LDH) **(B)** collected at baseline and following the match (post, 24h, 48h and 72 h) after cold water immersion (CWI) or thermoneutral water immersion (TWI). *Significant difference in comparison to baseline (*p* < 0.05). ^#^Significant difference in comparison to TWI (*p* < 0.05).

The increase in CK from baseline (100%) to post-match was significant, but not different between conditions (CWI, +76.5 ± 29.7%; TWI, +132.3 ± 70.4%, *p* > 0.05). Then, CK peaked at 24 h in both CWI (+154.5 ± 113%) and TWI (+216.5 ± 147.5%) conditions. CK returned to baseline values at 72 h in both conditions (Figure 4A). The increase in LDH from baseline (100%) to post-match was significant, and different between conditions at 48 h (CWI, 11.2 ± 11.3%; TWI, +25.4 ± 22.3%, *p* < 0.05, *d =* 0.8). Later, LDH recovered and returned to baseline values at 72 h in CWI but did not fully recover during the 72-h period in TWI (Figure 4B).

## 4 Discussion

The present study examined the impact of immediate post-exercise CWI following a simulated soccer match-play, compared to TWI (sham), on the time course of recovery of neuromuscular fatigue, physical performance and muscle damage biomarkers. The main data of this study indicated that the simulated soccer match-play resulted in considerable neuromuscular fatigue which persisted for up to 72 h post-exercise. The reduction in VA was statistically significant immediately after exercise and recovered at 24 h post-exercise. The reduction in Q_tw. pot_ was also significant immediately after exercise and remained depressed for up to 72 h post-exercise. The time course of neuromuscular fatigue was similar to findings reported after simulated (Thomas et al., 2017) or competitive soccer match-play (Brownstein et al., 2017). Even though previous studies investigating effects of CWI found that the soccer match-induced reduction in MVC (a ‘global’ index of neuromuscular fatigue) was attenuated through CWI (Bailey et al., 2007; Bouzid et al., 2018), they failed to discriminate if this form of cryotherapy affected central and/or peripheral components of fatigue. Here, using electrical nerve stimulations (Millet et al., 2011), we showed that both central and peripheral fatigue effects were substantially reduced immediately after CWI compared to TWI, leading to earlier full recovery of neuromuscular function and physical performance indices.

### 4.1 Effects of Post-match CWI on Peripheral Fatigue

When compared to TWI, CWI attenuated the development of post-match peripheral fatigue (Q_tw,pot_) by 9.1% at post-match and 8.6% at 72h, resulting in a faster recovery (Figure 2). Since M-waves were unaltered throughout recovery in the two experimental conditions, the greater peripheral fatigue in TWI was not linked to a reduced membrane excitability, and indicates that factors located beyond the sarcolemma, such as alterations in the excitation–contraction coupling, were responsible for the reduction in Q_tw,pot_ (Balog and Fitts, 1996).

The faster recovery of Q_tw,pot_ in CWI might be associated with a lower production of reactive oxygen species (ROS) and reactive nitrogen species (RNS) in the skeletal muscle, as they occur ∼3 days following intense exercise in mice (McArdle et al., 1999) and humans (Close et al., 2004). Indeed, an accumulation of ROS/RNS during exercise can affect the sarcolemma Ca^2+^ release, and has been associated with redox dependent modification of ryanodine receptors as well as a reduction in calcium sensitivity of myofilaments (Allen et al., 2008). Therefore, given the potential of CWI to contain inflammation and inhibit the production of ROS/RNS (White and Wells, 2013), it is likely that the faster CWI-induced recovery of peripheral fatigue following a simulated soccer match-play observed in this study is related to reduced ROS/RNS (Andrade et al., 1998). However, it is important to note that ROS/RNS were not collected in the present study and, hence, further explorations are required to confirm this assumption.

### 4.2 Effects of Post-Match CWI on Central Fatigue

In the present study, the interpolated twitch technique and VA calculation were used to assess central fatigue and its recovery kinetics (Merton, 1954). When compared to TWI, CWI attenuated post-match VA by 11.7% and accelerated its recovery (Figure 2). This result could be explained by the ability of CWI to reduce muscle damage, induced by the simulated soccer match-play. This ability was evidenced by the attenuated increase in LDH but, quite surprisingly, not in CK (Figure 4). However, similar discrepancies in blood markers were previously observed after CWI (Vaile et al., 2008). Overall, finding from previous studies investigating effects of CWI after a simulated soccer match-play (Bouzid et al., 2018) or jiu-jitsu training (Fonseca et al., 2016) confirm the positive effects of this therapy on muscle damage. The mechanism(s) responsible for the lower exercise-induced intracellular protein release to plasma following CWI remains unclear. Eston and Peters (1999) suggested that cryotherapy could reduce the post-exercise protein efflux from the muscle into the lymphatic system or reduce the amount of post-exercise damage. This indirect indication of lower muscle damage could be associated with decreased vessel permeability probably due to cryotherapy-induced attenuation of the inflammatory response (Eston and Peters, 1999). Interestingly, muscle damage, induced by prior eccentric exercise, was found to promote central fatigue during subsequent exercise (Endoh et al., 2005). Mechanistically, muscle damage/soreness stimulates the inhibitory feedback from group III/IV muscle afferents, through hormones involved in pain signaling such as bradykinin and prostaglandins (Kaufman et al., 1982), leading to increased central fatigue (i.e., reduction in VA). Similarly, greater ROS/RNS production in TWI compared to CWI, as discussed earlier, would also stimulate group III and IV muscle afferents (Delliaux et al., 2009). Finally, the direct implications of peripheral fatigue, in terms of promoting central fatigue by triggering group III and IV muscle afferent feedback (Amann et al., 2009; Hureau et al., 2018), may have contributed to the blunted VA in CWI compared to TWI.

Additionally, it has been shown that a passive (Morrison et al., 2004) or exercise-induced hyperthermia (Nybo and Nielsen, 2001) is associated with the development of central fatigue. Given the potential of CWI to reduce body temperature (Peiffer et al., 2010), it is likely that the attenuation of central fatigue observed following the CWI in the present study was also associated with a faster reduction in body temperature.

### 4.3 Recovery of Physical Performance

The present study showed that 20 m sprint performance was less impaired immediately after post-match intervention and throughout the time course of recovery in CWI compared to TWI (Figure 3), and this is consistent with previous investigations (Fonseca et al., 2016; Bouzid et al., 2018). It should be noted that post-match jump performances were not less impaired immediately in CWI compared to TWI, but they fully recovered 1 day earlier. Interestingly, the recovery kinetics of physical performance indices were slightly different than those of neuromuscular fatigue, indicating that performance is not a surrogate for fatigue measurement, and that other mechanisms than fatigue might explain the recovery of physical performance. A number of possible explanations could account for the discrepancies between recovery of neuromuscular fatigue and physical measures. Namely, there is an inherent level of variability associated with measures of voluntary performance, with individuals able to alter their jump or sprint mechanics in an attempt to maximize performance (Ratel et al., 2006). For example, it has previously been suggested that individuals alter their jump mechanics when fatigued in order to help maintain jump height (Gathercole et al., 2015). In addition, measures of neuromuscular function target the knee extensors under isometric conditions, while jump performance involves multi-joint dynamic movements, which could further contribute to the discrepancies. In the same line, a previous study found no correlation between the recovery of peripheral and central fatigue indices and physical performance indices following a competitive soccer match-play (Brownstein et al., 2017). Since CWI had a positive impact on the recovery of physical performance in the present study, it is highly important to emphasize that this impact was confirmed after a simulated, and not a competitive soccer match-play. Even so, this does not undermine the reliability of our reasoning, as we strongly believe that the simulated match-play adopted in this study had accurately simulated the physical demands of a competitive soccer match-play (Nicholas et al., 2000). Over and above that, in methodological terms, the use of this exercise modality had certainly contributed to a consistent simulation of physiological demands from one experimental visit to another (i.e., CWI vs. TWI); a crucial condition that unpredictable competitive soccer match-plays could have failed to achieve.

## 5 Practical Application

The present study showed that CWI, performed immediately after a simulated soccer match-play, blunted both central and peripheral components of neuromuscular fatigue. In turn, this resulted in a significant effect on subsequent sprint performance, which returned to pre-match values within 24 h following CWI, while it required a period of 72 h to fully recover following TWI. Given the value of sprints for performance in soccer (Haugen et al., 2014), CWI might serve as a powerful strategy to recover from a match in a congested competitive schedule context, i.e. with a second match in the same week. However, in training context, it is important to note that systematic/repetitive CWI after trainings sessions could led to lower training adaptations (Fröhlich et al., 2014) as it may restrain the activation of key proteins and satellite cells in the skeletal muscle (Roberts et al., 2015). Moreover, given the association between neuromuscular fatigue and muscle adaptations (Borresen and Lambert, 2009), the attenuation of fatigue following CWI in the present study may support the validity of previous evidence of the negative impact of CWI application on a chronic basis (Fröhlich et al., 2014; Roberts et al., 2015). Therefore, we recommend prescribing CWI only after matches, during an overcrowded competitive schedule, and not after training sessions.

CWI might be beneficial not only for physical performance, but also for reducing injury rates. Indeed, it has been documented that there is a six-fold increase in injury rate in elite soccer during the second match of the week (Dupont et al., 2010). Considering the importance of fatigue as an important injury risk factor (Eckard et al., 2018), a faster recovery in neuromuscular fatigue following CWI, as was shown in this study, is very likely to reduce injury rate. To confirm this idea, further longitudinal research is essential. Finally, the difference in time course of recovery between neuromuscular function and physical performance in the current study suggests that practitioners should use a range of both subjective and objective measures when monitoring fatigue and recovery in order to provide a more comprehensive understanding of readiness to train and compete.

Some limitations inherent to the experimental protocol of the study warrant mention. First, the number of participants was low due to the difficulty of the recruitment of professional soccer players. In fact, the protocol of the study entails several tests that many professional soccer players refused to undergo. Second, blood biomarkers of oxidative stress, which have the potential to stimulate the group III and IV muscle afferents, were not measured in our study. Finally, there is a significant cognitive component to a successful soccer performance that is not present in a simulated match, which could contribute to impairments in performance by increasing mental fatigue and the perception of effort required during a match (Smith et al., 2015). Further research is required with real soccer matches to further corroborate these findings in a more ecologically valid model.

## Conclusion

The findings of the present study showed that CWI immediately after a simulated soccer match-play significantly blunted both central and peripheral fatigue compared to TWI (sham), which led to earlier full recovery of neuromuscular function and physical performance indices. Therefore, CWI might be an interesting recovery strategy to consider, in particular in a congested schedule context, i.e., when there is a second match in the same week.

## Data Availability

The datasets presented in this article are not readily available because of privacy concerns. Requests to access the datasets should be directed to the corresponding author.
